# Thermodynamic Implications of Multiquintessence Scenario

**DOI:** 10.3390/e21090851

**Published:** 2019-08-31

**Authors:** Abdul Jawad, Zoya Khan, Shamaila Rani, Kazuharu Bamba

**Affiliations:** 1 Department of Mathematics, COMSATS University, Islamabad Lahore Campus, Lahore-54000, Pakistan; 2 Division of Human Support System, Faculty of Symbiotic, Systems Science, Fukushima University, Fukushima 960-1296, Japan

**Keywords:** multiquintessence, Hubble horizon, first-order formalism, thermodynamics, 95.36.+d, 98.80.–k

## Abstract

In this paper, we discuss the validity of the generalized second law of thermodynamics in the presence of a multi-component scalar field (ϕ) in a spatially flat Friedmann-Robertson-Walker (FRW) universe. We describe the first-order formalism by defining the Hubble parameter as $H=-W(\phi_{i})$. By using three super-potential models of the Hubble parameter, we analyze the validity of the generalized law and thermal equilibrium conditions in the presence of the logarithmically-corrected, Bekenstein-Hawking, Sharma-Mittal and $R\overset{´}{e}nyi$ entropies. It is noticed that the generalized law and thermal equilibrium conditions hold for some cases.

## 1. Introduction

In cosmology, many authors have considered the significance of the scalar field. To clarify the cosmic acceleration it may be connected to inflation which occurred in the early universe or as a dark energy (DE) candidate like quintessence [1,2,3]. Scalar fields may trigger various evolutionary scenarios of the universe’s expansion like the inflaton (inflationary era), the DE, the component of dark matter [4,5,6,7,8,9,10,11,12,13]. They all are characterized to be coupled to gravity, minimally or non-minimally [14,15,16,17,18]. In particular, in order to depict universe evolution, at least two scalar fields must collaborate over their kinetic or potential state. The quintom is the most basic multi-scalar field theory in which quintessence and phantom scalar fields attribute to DE portion of the region [19,20,21,22]. It is feasible to use just a single scalar field to interpret both DE and past inflation in quintessence inflation model [23]. Recently, to analyze the possible cosmic acceleration, quintessence is conjured up as an alternative of the cosmological constant [24]. For recent reviews on the DE problem including modified gravity theories, see, for example, References [25,26,27,28,29,30,31].

Roy and Bamba [32] explored interacting quintessence model with the quintessence potential, by utilizing the parametrization of interaction quintessence models, they extended the quintessence scalar field. Hertzberg et al. [33] investigated the fine-tuning of a quintessence model for dark energy in the framework of swampland conjectures. $D\overset{´}{i}az$ [34] restricted himself to the equation of state to study the problem of the quintessence potential, thus acquiring the statement of luminosity distance. By introducing a non-negative cosmological term they generalized the quintessence model, confining the scalar field energy density. Zlatev et al. [35] presented a form of quintessence tracker field. Including the new inspiration for the quintessence scheme, they demonstrated how it might clarify the occurrence. Roy and Banerjee [36] studied the dynamical system consideration of scalar fields. They checked for late time attractors and defined two examples, exponential and the power-law potentials. They examined the stable solutions for a few quintessence models. In the same context, Kleidis and Oikonomou [37] studied an f(R) theory in the presence of a canonical scalar field ϕ, by rendering the corresponding dynamical system autonomous. They accordingly showed that, in this case, the cosmological dynamical system is led to an unstable de Sitter attractor, which can be viewed as a graceful exit from inflation.

Scalar fields can also describe both late and early aspects of cosmic acceleration [38,39]. Yang et al. [40] considered different quintessence scalar field models and they found that for the early deceleration phase to the present cosmic acceleration all models carry out fine transition. They also found a strong negative relation among the parameters for all quintessence scalar field models. Shahalam et al. [41] studied the interaction of quintessence with scaling potential in spatially flat universe. They considered quintessence cosmological models as $\alpha {\dot{\rho }}_{m},\;\beta {\dot{\rho }}_{\phi }$ and $\sigma ({\dot{\rho }}_{m}+{\dot{\rho }}_{\phi }).$ They investigated the phase space analysis and dynamic behavior of these models and focused on the attractor solutions that can give rise to late time acceleration.

The connection to thermodynamic evolution is acquired by the idea of additional thermodynamical variables and entropy. Lymperis and Saridakis [42] utilized the tsallis entropy and through the application of the first law of thermodynamics (FLT) they constructed several cosmological scenarios. They showed that with the sequence of DE span and, depending on the value of the parameter of DE equation of state δ, during the evolution, experience the phantom-divide crossing and can be quintessence or phantom-like. Debnath et al. [43] investigated the equilibrium and non-equilibrium picture of the generalized second law of thermodynamics (GSLT) for event and apparent horizons for flat FRW metric which is filled with n-component fluid. In quintessence and phantom regimes they acquired constraints on the power-law parameter α. Bamba et al. [44] studied the GSLT in apparent and future event horizon in f(T) gravity. They also showed the conditions of the quintessence and phantom epoch in particular scenario by which GSLT will be valid. They also discussed validity of GSLT for logarithmic corrected entropy and power-law correction. Chakraborty and Guha [45] analyzed the validity of GSLT in an expanding Firedmann Walker Robertson (FRW) universe filled with different variants of Chaplygin gases. In the consequent part for the different models of the Chaplygin gas they investigated the validity of GSLT on the apparent and event horizons, they discovered that for these models the validity of GSLT on the event horizon depend on the choice of free parameter in the particular models. Tanisman et al. [46] checked the validity of GSLT for the D-dimensional Kaluza-Klein-type FRW universe in a thermal equilibrium state. Additionally, they demonstrated that their results can be reduces to lower dimensional cases in the restricting conditions.

We discuss the validity of GSLT in multiquintessence for the flat FRW metric. We organize paper as follows: In Section 2, we discuss the first-order formalism. In Section 3, we observe the behavior of GSLT and thermal equilibrium condition under various entropies. In Section 4, we summarize our results.

## 2. First-Order Formalism of Multi-Quintessence Scenario

We define the first-order formalism [47,48] for the coupled scalar fields with gravity. The action [49] of four-dimensional gravity is given in the form

(1) $$\begin{array}{c} S=\int d^{4}x\sqrt{\left|g\right|}\left[\frac{-R}{4}+\frac{1}{2}g_{ab}\nabla^{a}\phi_{i}\nabla^{b}\phi_{i}-V\left(\phi_{i}\right)\right], \end{array}$$

where *g* is the determinant of metric with signature (+,−,−,−), $d^{4}x$ is the invariant volume element in four dimensions, **R** stands for Ricci scalar, $\phi_{i},$i=1,2,…,N, defines real scalar fields, coupled to a set and $V\left(\phi_{i}\right)\equiv \left(\phi_{1},\phi_{2},\ldots ,\phi_{N}\right)$ is the potential which characterize the theory on the subject of a limited arbitrary number of scalar fields. The field equations for homogeneous and isotropic FRW metric is given by

(2) $$\begin{array}{ccc} H^{2} & = & \frac{8\pi G}{3}\rho -\frac{k}{a^{2}},\\ \dot{H}+H^{2} & = & -\frac{8\pi G}{6}\left(\rho +3p\right), \end{array}$$

where a(t) is the scale factor, *k* represents the curvature for flat (k=0), open (k=−1) and closed (k=1) spacetime, but here we only assume the flat case and ρ describes the energy density and *p* is the pressure of the system, respectively. The energy density and pressure are defined as [49]

(3) $$\begin{array}{ccc} \rho & = & \sum_{j=1}^{N}\frac{{\dot{\phi }}_{j}^{2}}{3}+V\left(\phi_{i}\right),\\ p & = & \sum_{j=1}^{N}\frac{{\dot{\phi }}_{j}^{2}}{3}-V\left(\phi_{i}\right). \end{array}$$

From the field equations, we have

$$\begin{array}{c} \dot{H}=-{\dot{\phi }}^{2}+\frac{k}{a^{2}}. \end{array}$$

Since scale factor a(t), scalar field ϕ(t) and Hubble parameter H(t) are functions of time and from Einstein’s equation we have to see the potential *V* as a function of time. We have potential V=V(ϕ) for the scalar field from the equation of motion, in this manner to make these two perspectives equivalent, we at that point need to see Hubble’s parameter as a function of ϕ. This is the key point and by introducing a new function W=W(ϕ), make it efficient. Now, we can understand that the *H* depends on time as a function of W[ϕ(t)] and we see that the potential *V* does not depend on the sign of W, thus the change W→−W leads to another possibility. The motivation for much of the above technique is based on former work [47], in which first-order formalism is used to examine first-order equations for at least two real scalar fields [50], up to arbitrary dimensions [51]. That is why one can choose first-order formalism as [47,48]

(4) $$\begin{array}{c} H=-W(\phi_{i}). \end{array}$$

For a flat FRW universe, Equation (4) leads to

(5) $$\begin{array}{c} \dot{\phi_{i}}=W_{\phi_{i}},\;i=1,2,\ldots ,N. \end{array}$$

The above Equations (4) and (5) allow us to write the potential term as follows

(6) $$\begin{array}{c} V\left(\phi_{i}\right)=\frac{3W{\left(\phi_{i}\right)}^{2}}{2}-\frac{1}{2}\sum_{i=1}^{N}W_{\phi_{i}}^{2}. \end{array}$$

where $\phi_{i}$ in subscript defines the derivative with respect to $\phi_{i}$, $W_{\phi_{i}}\equiv \frac{dW}{d\phi_{i}}$ and $\phi_{i}=\phi_{i}\left(t\right).$ From Equation (1), the equation of motion for scalar fields is defined as

(7) $$\begin{array}{c} {\ddot{\phi }}_{i}+3H{\dot{\phi }}_{i}+V_{\phi_{i}}=0, \end{array}$$

where $V_{\phi_{i}}\equiv \frac{dV}{d\phi_{i}}$ and double dot represents derivative with respect to time.

We consider the three models of Hubble parameter and the corresponding solutions of scalar fields. The models are:

### 2.1. Model 1

First, we choose the superpotential, that is, $Z_{2}$ model [52] and the direct sum of sine-Gordon [53], given by

(8) $$\begin{array}{c} W\left(\phi_{1},\phi_{2}\right)=\lambda_{1}\left(\phi_{1}-\frac{\phi_{1}^{3}}{3}\right)+\lambda_{2}\sinh\left(\phi_{2}\right)+\alpha_{1}, \end{array}$$

where $\lambda_{1}$, $\lambda_{2}$ and $\alpha_{1}$ are arbitrary constants. The motivation to research this model (mixture of $Z_{2}$ and sine-Gordon models), begins from the way that those are found in numerous areas of Physics, including condensed matter physics, field theory and cosmology among many others [49]. Table 1, shows the first-order equations and corresponding solutions of scalar fields.

### 2.2. Model 2

The second superpotential is the combination of sine-Gordon, $Z_{2}$ and BNRT models [49,54], we have

(9) $$\begin{array}{c} W\left(\phi_{1},\phi_{2},\phi_{3},\phi_{4}\right)=\lambda_{1}\left(\phi_{1}-\frac{\phi_{1}^{3}}{3}\right)+\lambda_{2}\sinh\left(\phi_{2}\right)\\ -\lambda_{3}\phi_{3}+\frac{\lambda_{3}\phi_{3}^{3}}{3}+\mu_{3}\phi_{3}\phi_{4}^{2}+\alpha_{2}, \end{array}$$

The first-order equations and solutions of $\phi_{1}$ and $\phi_{2}$ are same as in model 1. The BNRT model [54] is utilized for modeling a great number of systems [55,56,57,58,59]. By using the so-called configurational entropy, the rich structure of this model permits the generation of double-kink configuration [60], as shown in Reference [61]. In Table 2, we show the differential equations and their solutions.

### 2.3. Model 3

For third model [49,62] the superpotential is as follows

(10) $$\begin{array}{c} W\left(\phi_{1},\phi_{2},\phi_{3},\phi_{4}\right)=\lambda_{1}\left(\phi_{1}-\frac{\phi_{1}^{3}}{3}\right)+\lambda_{2}\sin\left(\phi_{2}\right)-\frac{\lambda_{3}\phi_{3}^{3}}{3}\\ -\phi_{3}^{2}\phi_{4}+\phi_{4}-\frac{\phi_{4}^{3}}{3}+\alpha_{3}, \end{array}$$

where $\alpha_{3}$ is arbitrary constant. The first-order differential equations and solutions of $\phi_{1}$ and $\phi_{2}$ are of model 1. The dependence of $\phi^{3}-\phi^{4}$ was inspired in a model examined by Brito and Dutra [62], where they found interesting analytical solutions of asymmetric two-kink. Table 3, shows the first-order differential equations and solutions of the equations which satisfied the differential equations.

## 3. GSLT and Thermal Equilibrium Condition

Inspired by black hole (BH) thermodynamics [63], it was realized that there should be a connection between gravity and thermodynamics. For this purpose, Jacobson [64] derived a relation between thermodynamics and the Einstein field equations on the basis of entropy-horizon area proportionality relation along with first law of thermodynamics dQ=TdS. Here dQ,T and dS indicate the exchange in energy, temperature and entropy change for a given system. It was found [65] that the field equations can be expressed in terms of TdS=dE+pdV (E,p and *V* represent the internal energy, pressure and volume of the spherical system) for any spherically symmetric spacetime in any horizon. It is a well-established phenomenon that our universe undergoes accelerated expansion in the presence of DE. A question arises about the thermodynamical behavior of the universe experiencing accelerated expansion, in particular, what is the fate of GSLT in this scenario? We provide a review of GSLT for a system containing a BH. Basically, GSLT for a cosmological system is the generalization of GSLT for a system containing a BH as proposed by Bekenstein [66]. Bekenstein argued that the common entropy in the BH exterior plus the BH entropy never decreases. This statement is based on the proportionality relation between entropy of BH horizon and horizon area.

In next sections, we study the validity of GSLT of the multiquintessence at Hubble horizon for a flat FRW universe. We find the Hubble horizon calculated by the condition [67,68] $h^{\mu \nu }\partial_{\mu }R_{A}\partial_{\nu }R_{A}=0$, this condition gives the Hubble horizon as

$$R_{A}=\frac{1}{H}.$$

From the above expression, we get

(11) $$\begin{array}{c} dR_{A}=HR_{A}^{3}\left(\rho +p\right)dt. \end{array}$$

The Bekenstein entropy is defined as $S=\frac{A}{4G},$ where $A=4\pi r^{2}$ is the area of the horizon [66,69]. By using the Bekenstein entropy, Equation (11) becomes

(12) $$\begin{array}{c} \frac{G}{2\pi R_{A}}dS=HR_{A}^{3}\left(\rho +p\right)dt. \end{array}$$

The temperature of the horizon is defined as [70]

(13) $$\begin{array}{ccc} T_{h}=\frac{|k_{sg}|}{2\pi },\;\;k_{sg} & = & \frac{1}{2\sqrt{-h}}\partial_{\mu }\left(\sqrt{-h}h^{\mu \nu }\partial_{\nu }R_{A}\right)\\ & = & -\frac{1}{R_{A}}\left(1-\frac{{\dot{R}}_{A}}{2HR_{A}}\right)=-\frac{R_{A}}{2}\left(2H^{2}+\dot{H}\right). \end{array}$$

Multiplying $T_{h}=-\frac{1}{2\pi R_{A}}\left(1-\frac{\dot{R_{A}}}{2HR_{A}}\right)$ on both sides of Equation (12), we have

(14) $$\begin{array}{c} T_{h}dS=\left[-4\pi HR_{A}^{3}dt+2\pi R_{A}^{2}dR_{A}\right]\left(\rho +p\right). \end{array}$$

Now, we define the Misner-sharp energy which is $E=\frac{R_{A}}{4G},$ we describe the energy density in terms of the volume $V=\frac{4\pi R_{A}^{3}}{3},$ which becomes

(15) $$\begin{array}{c} \tilde{E}=\frac{3H^{2}}{8\pi G}V\equiv \rho V. \end{array}$$

By taking the differential of the energy density, we easily find

(16) $$\begin{array}{c} d\tilde{E}=-4\pi HR_{A}^{3}\left(\rho +p\right)dt+4\pi R_{A}^{2}\rho dR_{A}. \end{array}$$

Assembling Equations (14) and (16), we can obtain

(17) $$\begin{array}{c} T_{h}dS=d\tilde{E}-2\pi R_{A}^{2}\left(\rho -p\right)dR_{A}. \end{array}$$

The work density is defined as

(18) $$\begin{array}{c} \tilde{W}=-\frac{1}{2}\left(T^{(M)\mu \nu }h_{\mu \nu }+{\tilde{T}}^{(de)\mu \nu }h_{\mu \nu }\right)=\frac{1}{2}\left(\rho -p\right), \end{array}$$

utilizing the work density in Equation (17), we get

(19) $$\begin{array}{c} T_{h}dS=d\tilde{E}-\tilde{W}dV. \end{array}$$

Equation (19) shows that first law of thermodynamics (FLT) is satisfied in multi-component scalar field.

However, GSLT states that sum of the black hole entropy and the entropy of the black hole external region can never be decreased [71,72]. The condition that satisfies the entropy relation, described as

(20) $$\begin{array}{c} {\dot{S}}_{tot}=\dot{S}+{\dot{S}}_{in}\geq 0, \end{array}$$

where ${\dot{S}}_{tot}$ is the total entropy of the energy and matter inside the horizon, $\dot{S}$ relates to the horizon entropy and ${\dot{S}}_{in}$ correspond to the inner horizon with the sum of all entropy components. To study the GSLT, we now proceed with modified FLT,

(21) $$\begin{array}{c} T_{i}dS_{i}=dE_{i}+p_{i}dV, \end{array}$$

which can be written as

(22) $$\begin{array}{c} T_{in}{\dot{S}}_{in}=4\pi R_{A}^{2}\left({\dot{R}}_{A}-HR_{A}\right)\left(\rho_{i}+p_{i}\right). \end{array}$$

$T_{in}$ denotes the temperature of the inner horizon for all components. Here $\sum_{i}\left(\rho^{i}+p^{i}\right)=\rho +p,$ the total entropy inside the horizon becomes

(23) $$\begin{array}{c} T_{in}dS_{in}=4\pi R_{A}^{2}\left({\dot{R}}_{A}-HR_{A}\right)\left(\rho +p\right). \end{array}$$

Taking the time derivative of Bekenstein entropy, we find out

(24) $$\begin{array}{c} \dot{S}=-\frac{2\pi \dot{H}}{H^{3}G} \end{array}$$

The thermal equilibrium set with $T_{in}=T_{h}$ and Equation (23) leads to

(25) $$\begin{array}{c} {\dot{S}}_{in}=\frac{1}{T_{h}}\left(\rho +p\right)4\pi R_{A}^{2}\left({\dot{R}}_{A}-HR_{A}\right). \end{array}$$

After some calculations, it results

(26) $$\begin{array}{c} {\dot{S}}_{in}=\frac{4\pi }{G}\left[\frac{\dot{H}\left(\dot{H}+H^{2}\right)}{H^{3}\left(\dot{H}+2H^{2}\right)}\right]. \end{array}$$

By putting Equations (24) and (26) in (20), we get

(27) $$\begin{array}{c} {\dot{S}}_{tot}=\frac{\pi }{W^{2}}\left[\frac{16\pi W_{\phi }^{2}\left(-W_{\phi }^{2}+W^{2}\right)}{W(-W_{\phi }^{2}+2W^{2})}-\frac{2W_{\phi }^{2}}{W}\right]. \end{array}$$

**Figure 1:**
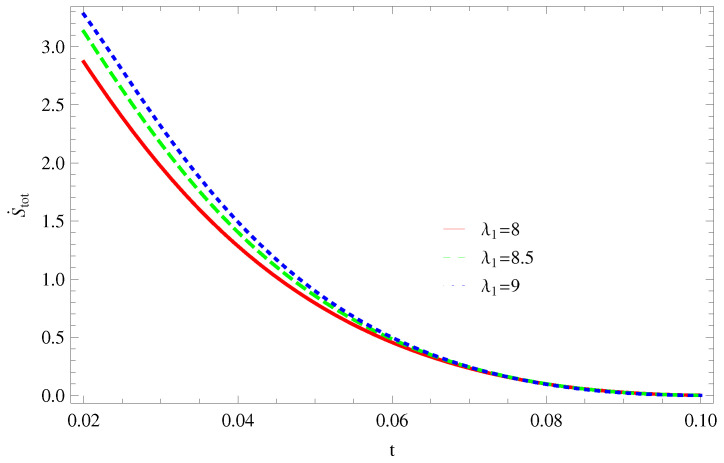
Plot of ${\dot{S}}_{tot}$ versus *t* for model 1.

**Figure 2:**
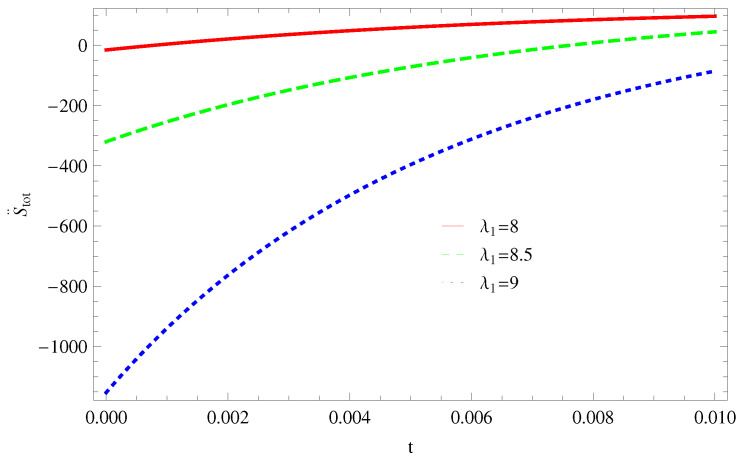
Plot of ${\ddot{S}}_{tot}$ versus *t* for model 1.

**Figure 3:**
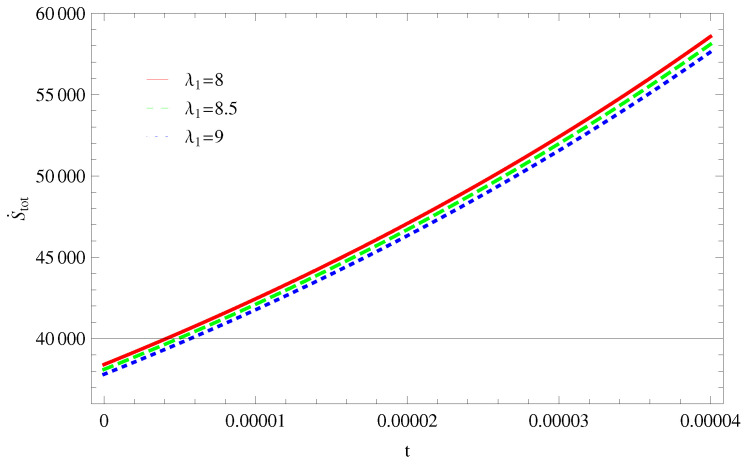
Plot of ${\dot{S}}_{tot}$ versus *t* for model 2.

**Figure 4:**
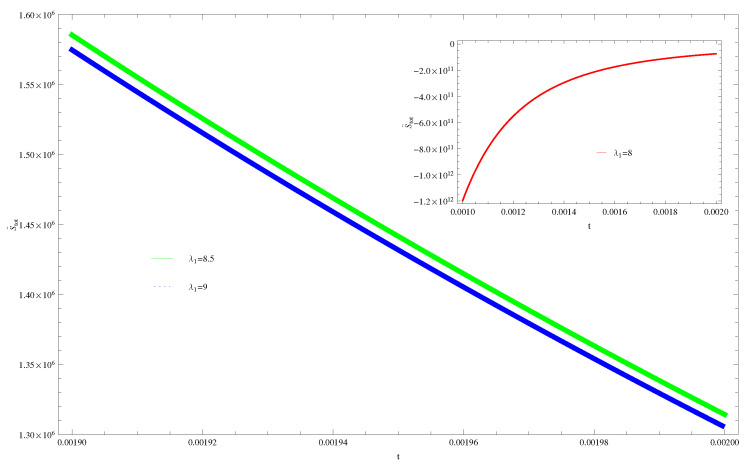
Plot of ${\ddot{S}}_{tot}$ versus *t* for model 2.

**Figure 5:**
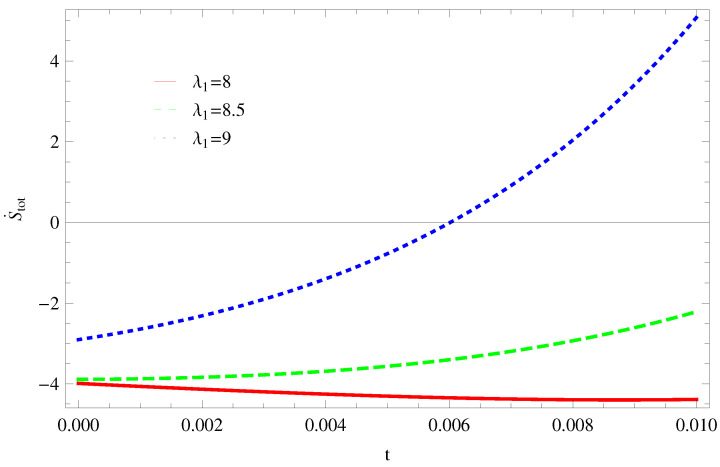
Plot of ${\dot{S}}_{tot}$ versus *t* for model 3.

**Figure 6:**
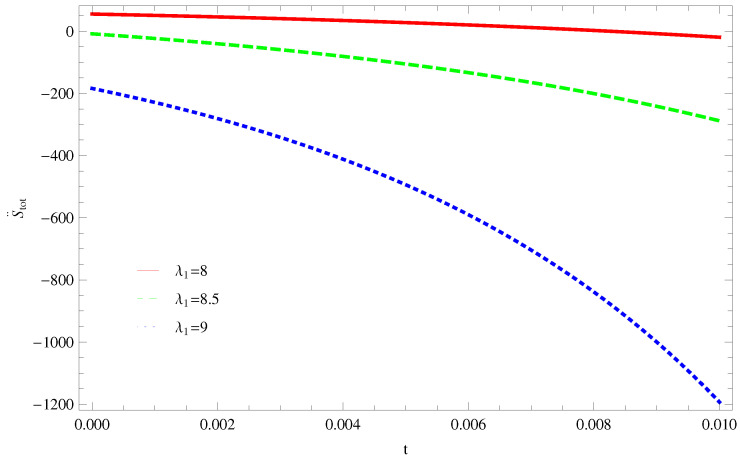
Plot of ${\ddot{S}}_{tot}$ versus *t* for model 3.

- For Model 1:
  In Figure 1, we plot graph of ${\dot{S}}_{tot}$ versus time for Bekenstein entropy at Hubble horizon for flat spacetime. We choose values of parameter $\lambda_{1}=8,8.5,9,\;\lambda_{2}=-4,\;\alpha =5.$ All trajectories are decreasing positively with the increasing value of t, which shows the validity of GSLT. In Figure 2, for $\lambda_{1}=8$, the trajectory is initially negative, while showing a transition towards negative direction after some epoch. It means thermal condition holds at the present as well as early epoch but remains invalid in the later epoch. However, the trajectories remain in the negative phase which exhibits the validity of the thermal condition for $\lambda_{1}=8.5,9$.
- For Model 2:
  Figure 3, shows the graph of ${\dot{S}}_{tot}$ versus t. With the same values of $\lambda_{1},\;\lambda_{2}$, $\lambda_{3}=5,\;c=5,\;\mu =5.5$ and α=4. All trajectories are gradually increasing in a positive direction at the present epoch as well as the later epoch with the increasing value of *t* which leads to the validity of GSLT. Figure 4, shows that for $\lambda_{1}=8$ the trajectory remains in a negative phase at a later epoch which fulfils the thermal condition and for $\lambda_{1}=8.5,9$, trajectories show decreasing behavior towards positive direction at a later epoch and cannot maintain the stability of thermal condition.
- For Model 3:
  By taking the same values of $\lambda_{1},\;\lambda_{2},\;\lambda_{3},\;c$ and α=−6.5, Figure 5, demonstrate that for $\lambda_{1}=9$, GSLT preserved the validity in the later epoch while remains invalid for other two cases of $\lambda_{1}=8,8.5$. Figure 6 shows that the thermal equilibrium condition at the later epoch for $\lambda_{1}=8$. However, thermal stability occurs for two other cases $\lambda_{1}=8.5,9$ at the present epoch as well as the later epoch.

### 3.1. Sharma-Mittal Entropy

The unique entropy measure is the Sharma-Mittal entropy [73,74] that permits a rise to a thermostatistics. We here introduce this entropy to discuss the validity of GSLT, which is

(28) $$\begin{array}{c} S_{SM}=\frac{1}{1-r}\left[{\left(1+\frac{\delta A}{4}\right)}^{\frac{1-r}{\delta }}-1\right], \end{array}$$

where *r* is the free parameter. By taking the time derivative of above entropy, we have

(29) $$\begin{array}{c} {\dot{S}}_{SM}=2\pi R_{A}{\dot{R}}_{A}{\left(1+\delta \pi R_{A}^{2}\right)}^{\frac{1-r}{\delta }-1}. \end{array}$$

For the case of apparent horizon, Equation (29) becomes

(30) $$\begin{array}{c} {\dot{S}}_{SM}=-\frac{2\pi \dot{H}}{H^{3}}{\left(1+\frac{\delta \pi }{H^{2}}\right)}^{\frac{1-r}{\delta }-1}. \end{array}$$

Inserting Equations (26) and (30) in (20), we have

(31) $$\begin{array}{c} {\dot{S}}_{tot}=\frac{\pi }{W^{2}}\left[\frac{16\pi W_{\phi }^{2}\left(-W_{\phi }^{2}+W^{2}\right)}{W(-W_{\phi }^{2}+2W^{2})}-\frac{2W_{\phi }^{2}}{W}{\left(1+\frac{\delta \pi }{W^{2}}\right)}^{\frac{1-r}{\delta }-1}\right] \end{array}$$

We check the validity of GSLT and thermal equilibrium condition for Equation (31) at Hubble horizon by a graphical representation.

**Figure 7:**
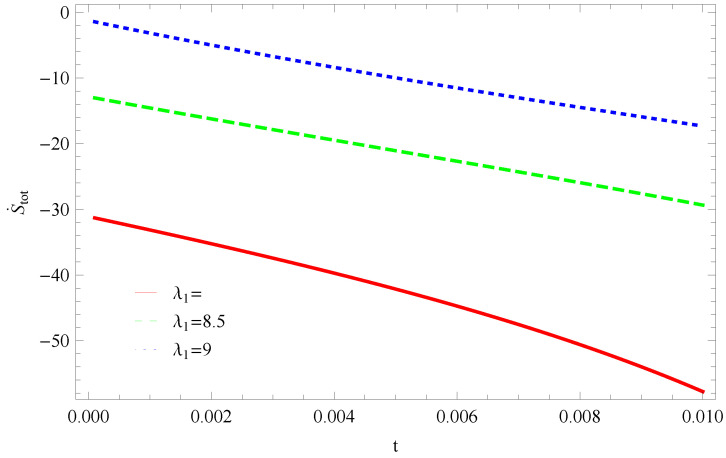
Plot of ${\dot{S}}_{tot}$ versus *t* for model 1.

**Figure 8:**
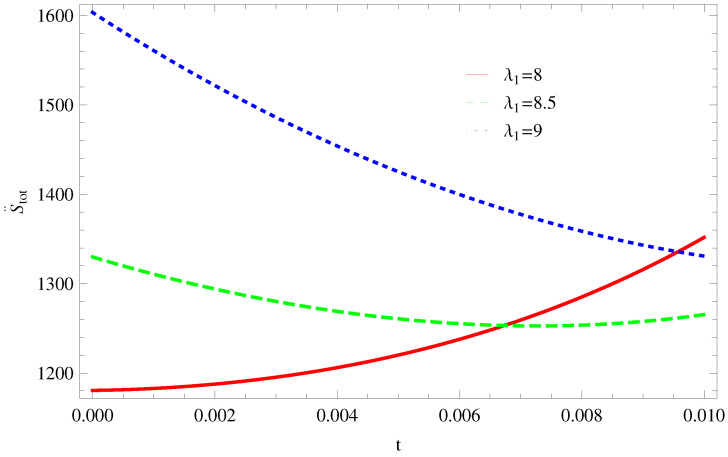
Plot of ${\ddot{S}}_{tot}$ versus *t* for model 1.

**Figure 9:**
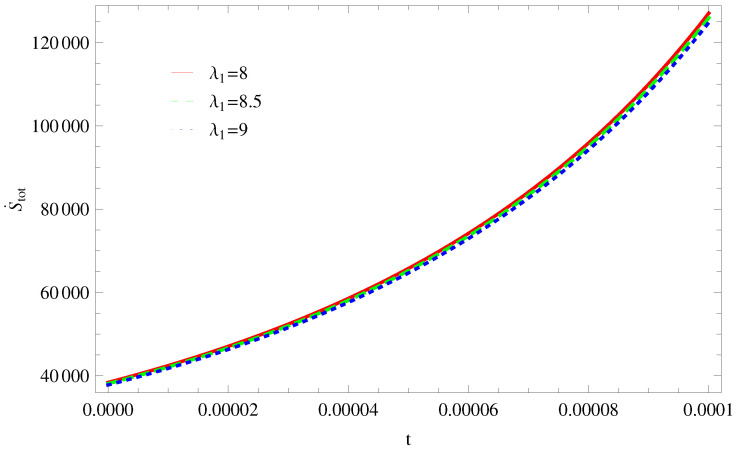
Plot of ${\dot{S}}_{tot}$ versus *t* for model 2.

**Figure 10:**
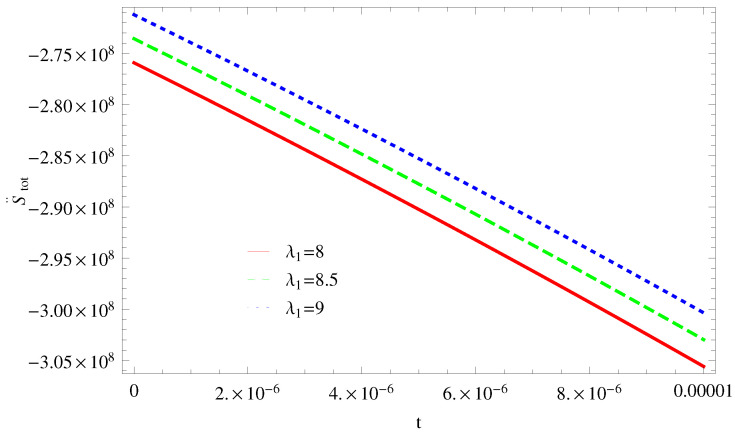
Plot of ${\ddot{S}}_{tot}$ versus *t* for model 2.

**Figure 11:**
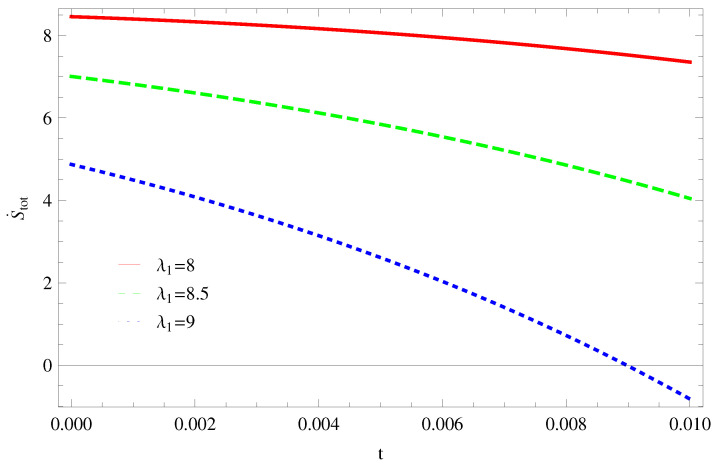
Plot of ${\dot{S}}_{tot}$ versus *t* for model 3.

**Figure 12:**
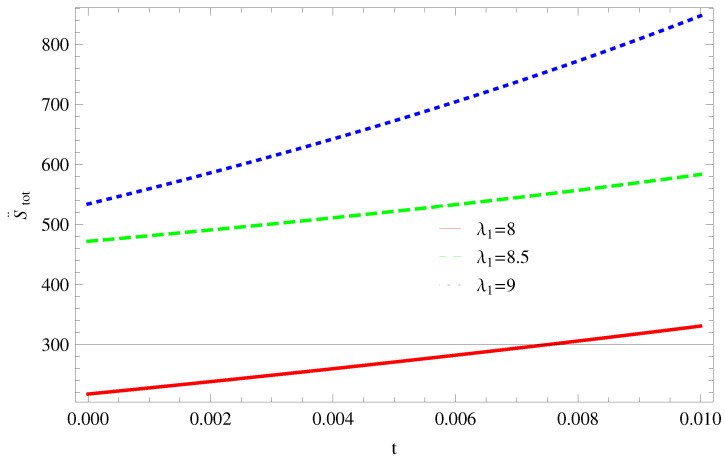
Plot of ${\ddot{S}}_{tot}$ versus *t* for model 3.

- For Model 1:
  In Figure 7, by taking the same values of $\lambda_{1},\;\lambda_{2},\;\alpha$ and r=1,δ=0.1, the graph demonstrates that all trajectories gradually decreasing towards negative direction and remain in the negative phase at later epoch, which cannot fulfills the stability condition for GSLT. With the same values of $\lambda_{1},\;\lambda_{2},\;\alpha ,\;r$ and δ and $\lambda_{1}=8,\;8.5,\;9$, the thermal stability remain invalid as all trajectories remain in a positive phase at the present epoch as well as the later epoch Figure 8.
- For Model 2:
  All trajectories are increasing in a positive direction at the present epoch as well as the later epoch with the increasing value of *t*. By taking the same values of all parameters $\lambda_{1},\;\lambda_{2},\;\lambda_{3},\;r,\;c,\;\mu ,\;\alpha ,$ which confirms the validity of GSLT (Figure 9). In Figure 10, the thermal equilibrium condition satisfies the same values of all parameters as all trajectories for $\lambda_{1}=8,8.5,9$ remain in the negative phase at present as well as in the later epoch.
- For Model 3:
  With the same values of all parameters, the left trajectories (Figure 11) show that for $\lambda_{1}=8,8.5$ it remains in the positive phase and for $\lambda_{1}=9$ it gradually decreases towards a negative phase at a later epoch. Thus, the validity of GSLT confirms only for two cases $\lambda_{1}=8,8.5.$ As we increase the value of $\lambda_{1}$ the stability condition cannot maintain. The right side trajectory $\lambda_{1}=8$ increasing towards a positive phase at present as well as the later epoch (Figure 12) and for $\lambda_{1}=8.5,9$, trajectories show increasing behavior at the later epoch and remain in the positive phase which cannot preserve the thermal condition.

### 3.2. Logarithmic Corrected Entropy

The entropy-area relation including quantum corrections conduct curvature corrections within Einstein-Hilbert action. The Bekenstein-Hawking logarithmic corrected entropy is defined by the relation

(32) $$\begin{array}{c} S=\frac{A}{4G}+\alpha \ln\frac{A}{4G}+\beta \frac{4G}{A}+\gamma , \end{array}$$

where α,β and γ are dimensionless constants, apart from the exact values of these constants are still checked. The thermodynamics of BH is modified by the thermal fluctuations and emerge as more important for smaller size BHs with adequately high temperature. In this mode, entropy can be modified as above. Now, by taking the time derivative of logarithmic corrected entropy, we easily get

(33) $$\begin{array}{c} \dot{S}=\left(\frac{\dot{A}}{A}\right)\frac{A}{4G}\left[1+\alpha \frac{4G}{A}-\beta {\left(\frac{4G}{A}\right)}^{2}\right]. \end{array}$$

In the case of apparent horizon, Equation (33) becomes

(34) $$\begin{array}{c} \dot{S}=-\frac{2\pi \dot{H}}{H^{3}}\left[1+\alpha \frac{GH^{2}}{\pi }-\beta (\frac{GH^{2}}{\pi }{)}^{2}\right]. \end{array}$$

Putting the values of ${\dot{S}}_{in}$ and $\dot{S}$ in Equation (20), we have

(35) $$\begin{array}{c} {\dot{S}}_{tot}=\frac{\pi }{W^{2}}\left[\frac{16\pi W_{\phi }^{2}\left(-W_{\phi }^{2}+W^{2}\right)}{W(-W_{\phi }^{2}+2W^{2})}-\frac{2W_{\phi }^{2}}{W}\left[1+\alpha \frac{W^{2}}{4\pi^{2}}-\beta {\left(\frac{W^{2}}{4\pi^{2}}\right)}^{2}\right]\right]. \end{array}$$

Equation (35) graphically representing to observe the validity of GSLT and thermal equilibrium condition at Hubble horizon.

**Figure 13:**
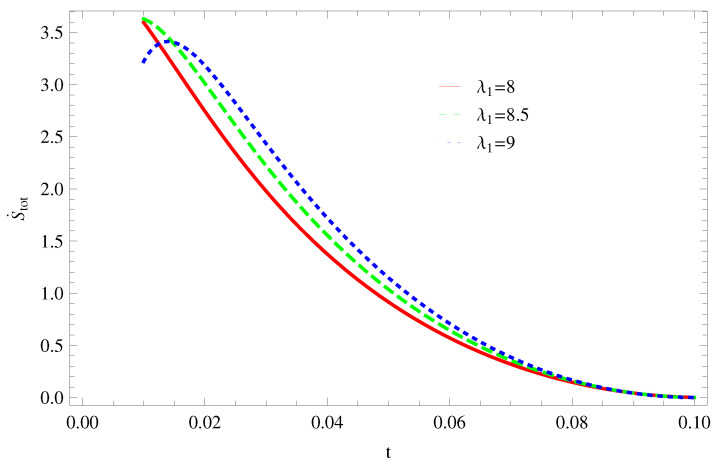
Plot of ${\dot{S}}_{tot}$ versus *t* for model 1.

**Figure 14:**
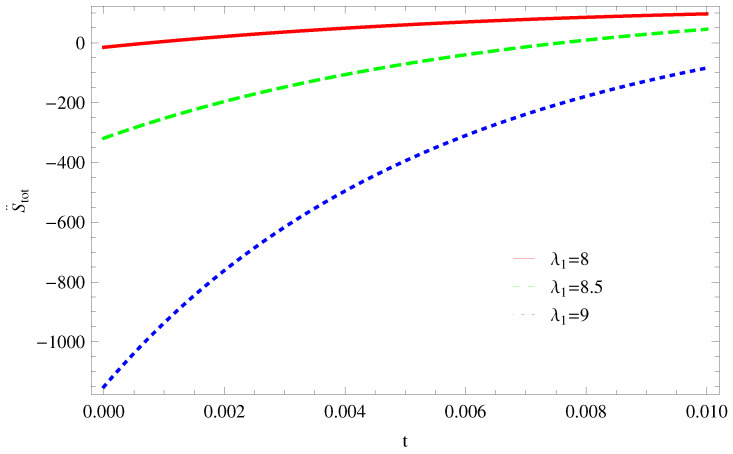
Plot of ${\ddot{S}}_{tot}$ versus *t* for model 1.

**Figure 15:**
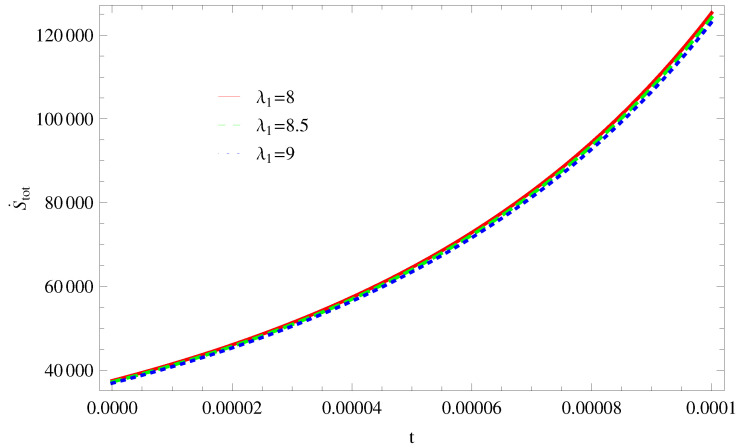
Plot of ${\dot{S}}_{tot}$ versus *t* for model 2.

**Figure 16:**
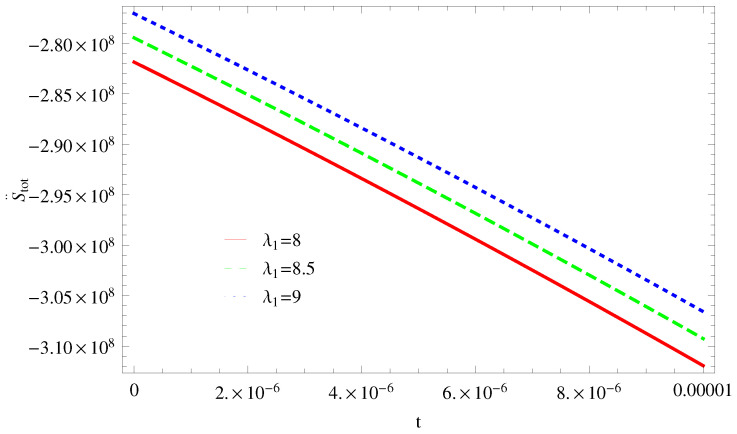
Plot of ${\ddot{S}}_{tot}$ versus *t* for model 2.

**Figure 17:**
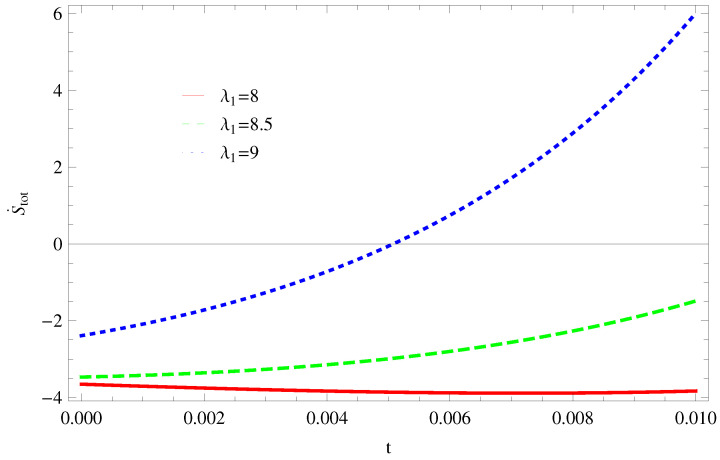
Plot of ${\dot{S}}_{tot}$ versus *t* for model 3.

**Figure 18:**
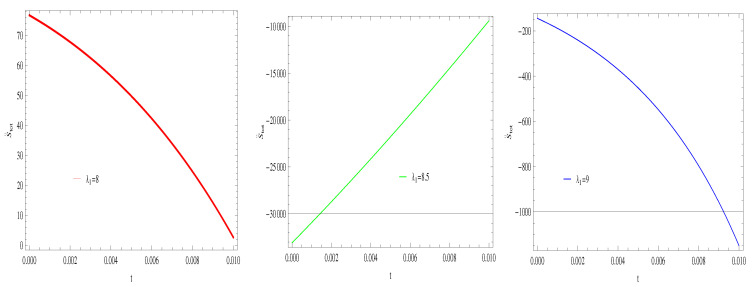
Plot of ${\ddot{S}}_{tot}$ versus *t* for model 3.

- For Model 1:
  In Figure 13, with the same values of $\lambda_{1},\;\lambda_{2},\;\alpha$ and $\alpha_{1}=2,\;\beta =1.5$, the trajectories decreasing towards positive direction at later epoch with the increasing value of t, which shows the validity of GSLT. The trajectories of (Figure 14) show that for $\lambda_{1}=8$ trajectory increasing in positive phase at present epoch as well as later epoch. For $\lambda_{1}=8.5,9$ the trajectory remains in the negative phase at present epoch and later epoch. Hence, with the increasing value of $\lambda_{1}$ the stability condition maintain and the thermal condition satisfies.
- For Model 2:
  The left side trajectories increasing positively at present epoch as well as later epoch with the increasing values of *t*, for all constant parameters, which shows that the GSLT is valid (Figure 15). With the same values of all parameters, the thermal equilibrium condition satisfies, as all trajectories decreases at present as well as the later epoch and remain in the negative phase for $\lambda_{1}=8,8.5,9$ (Figure 16).
- For Model 3:
  Figure 17, demonstrate that by taking the same values of $\lambda_{1},\lambda_{2},\lambda_{3},c$ and α=−6.5, for $\lambda_{1}=9$, GSLT preserved the validity in the later epoch while remains invalid for other two cases of $\lambda_{1}=8,8.5$. In Figure 18, we plot three graphs for $\lambda_{1}=8,8.5,9,$ in first graph for $\lambda_{1}=8$ shows that trajectory decreasing towards positive direction at present as well as later epoch, for $\lambda_{1}=8.5$ the trajectory is remain in negative phase at present epoch as well as later epoch and for $\lambda_{1}=9$ the trajectory decreasing negatively. Hence, the thermal equilibrium condition satisfies for $\lambda_{1}=8.5,9$ and is invalid for the case $\lambda_{1}=8.$

### 3.3. Rényi Entropy

Recently Rényi generalized entropy has been extensively used in order to study various gravitational and cosmological frameworks. The Rényi entropy is also important in quantum information where it can be used as a measure of tangle [75]. Here we define Rényi entropy to check the validity of GSLT, we have

(36) $$\begin{array}{c} S=\frac{1}{\delta }\ln\left(1+\frac{\delta A}{4}\right), \end{array}$$

where δ=1−Q, time derivative of Equation (36) is

(37) $$\begin{array}{c} \dot{S}=\frac{2\pi \delta R_{A}{\dot{R}}_{A}}{\delta (1+\delta \pi R_{A}^{2})}, \end{array}$$

Rényi entropy written in apparent horizon case as

(38) $$\begin{array}{c} \dot{S}=-\frac{2\pi \dot{H}}{H(H^{2}+\delta \pi )}, \end{array}$$

with given entropy Equation (20) becomes

(39) $$\begin{array}{c} {\dot{S}}_{tot}=16\pi^{2}\left[\frac{W_{\phi }^{2}\left(-W_{\phi }^{2}+W^{2}\right)}{W^{3}\left(-W_{\phi }^{2}+2W^{2}\right)}\right]-\frac{2\pi W_{\phi }^{2}}{W(W^{2}+\delta \pi )}. \end{array}$$

Form Equation (39) we check the validity of GSLT and thermal equilibrium condition for Rényi entropy at Hubble horizon.

**Figure 19:**
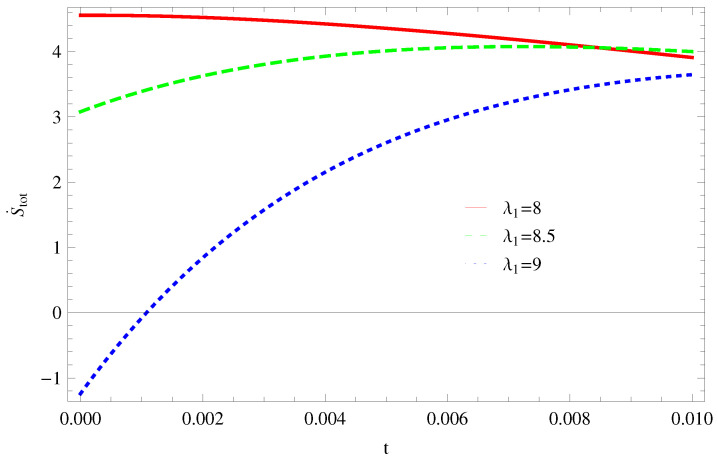
Plot of ${\dot{S}}_{tot}$ versus *t* for model 1.

**Figure 20:**
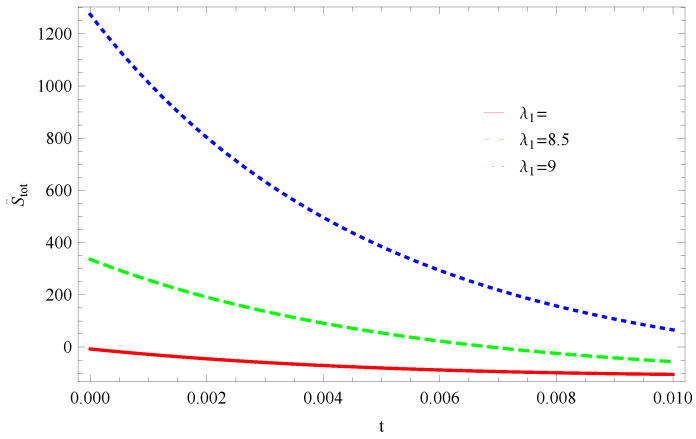
Plot of ${\ddot{S}}_{tot}$ versus *t* for model 1.

**Figure 21:**
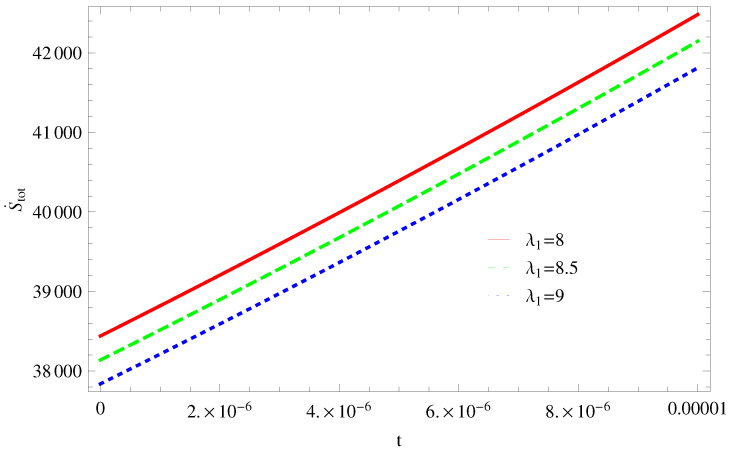
Plot of ${\dot{S}}_{tot}$ versus *t* for model 2.

**Figure 22:**
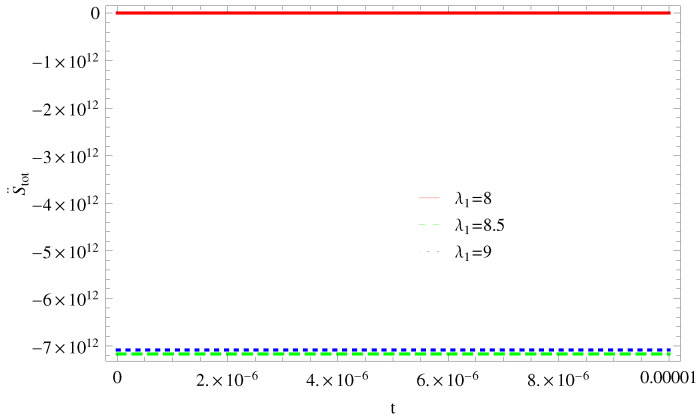
Plot of ${\ddot{S}}_{tot}$ versus *t* for model 2.

**Figure 23:**
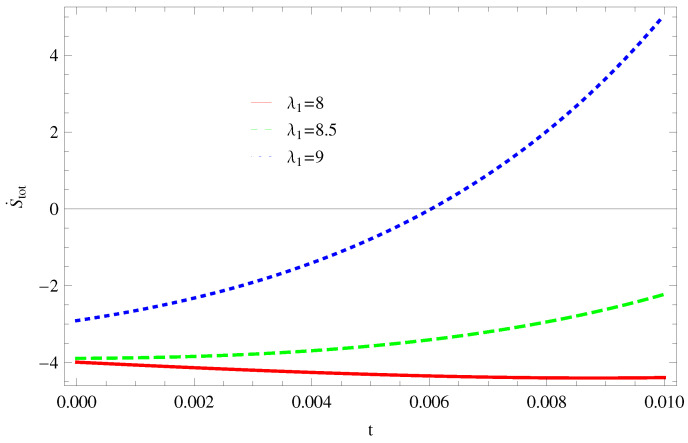
Plot of ${\dot{S}}_{tot}$ versus *t* for model 3.

**Figure 24:**
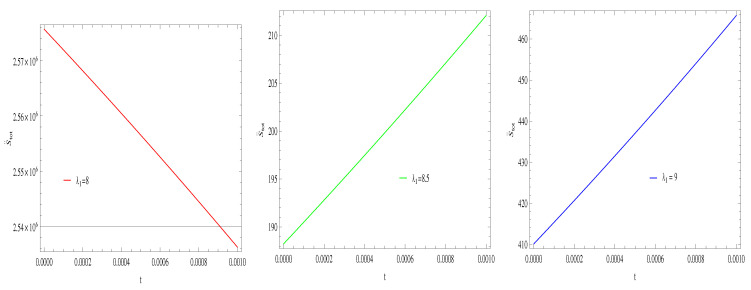
Plot of ${\ddot{S}}_{tot}$ versus *t* for model 3.

- For Model 1:
  Figure 19, shown that the trajectory decreases in positive phase for $\lambda_{1}=8$ and for other two cases $\lambda_{1}=8.5,\;9$ trajectories increases toward positive direction at present as well as later epoch, which confirms the validity of GSLT. In Figure 20, the trajectories for $\lambda_{1}=8,\;8.5,\;$ gradually decreases at present epoch as well as later epoch and preserved the thermal condition and for the decreasing trajectory in the positive phase for $\lambda_{1}=9$ the stability condition cannot maintain and the thermal equilibrium condition is invalid for this case.
- For Model 2:
  In Figure 21, by taking the same values of $\lambda_{1},\;\lambda_{2},\;\lambda_{3},\;\alpha ,\;c,\;\mu$ and δ, for all cases of $\lambda_{1}$ all trajectories remain in the positive phase at present as well as later epoch which demonstrate that the GSLT is valid. In Figure 22, the trajectory remain constant in positive phase $\left(\lambda_{1}=8\right)$ which cannot preserved the thermal condition and for other two cases $\left(\lambda_{1}=8.5,9\right)$ trajectories remain constant in negative phase which confirms the thermal condition.
- For Model 3:
  Figure 23, for $\lambda_{1}=9$, GSLT preserved the validity in the later epoch while remains invalid for other two cases of $\lambda_{1}=8,8.5,$ by taking the same values of all parameters. Figure 24, demonstrate that with the same values of all parameters the thermal condition cannot satisfies for all cases of $\lambda_{1}$ at present epoch as well as later epoch as all trajectories remain in the positive phase.

## 4. Summary

We examined the validity of GSLT in multiquintessence at Hubble horizon for flat FRW universe. Throughout we follow the first-order formalism and choose the Hubble parameter $H=-W(\phi_{i}).$ We take three different superpotential models of the Hubble parameter. Model 1 is the direct sum of $Z_{2}$ and sine-Gordon, Model 2 is the combination of $Z_{2},$ BNRT and sine-Gordon and in Model 3 we consider the modified BNRT model along with $Z_{2}$ and sine-Gordon models. We also check the stability condition of the thermal equilibrium and GSLT at present epoch and later epoch for Bekenstein entropy at Hubble horizon along with three different Hubble parameter models for flat spacetime. We also choose three different entropies as an example which are Sharma-Mittal, logarithmic corrected and Rényi entropies, to observed the stability condition for each models of the $W(\phi_{i})$ at Hubble horizon for flat spacetime. We also analyzed that FLT satisfied in multiquintessence at Hubble horizon for flat spacetime. To checked the stability condition we plot graphs by taking the same values of all parameters and for different cases of $\lambda_{1}$ by choosing three different values for flat spacetime along with different $W(\phi_{i})$ in the constructed model of each entropy at Hubble horizon.

The stability of GSLT validity and thermal condition at Hubble horizon for flat spacetime with all entropies are given below:

- For Bekenstein entropy
  For Model 1 and Model 2 the stability condition preserved the validity of GSLT for Bekenstein entropy and thermal equilibrium condition only satisfied for $\lambda_{1}=9$ for Model 1 and in Model 2 the stability condition for thermal equilibrium satisfied only for $\lambda_{1}=8$ at later epoch, while for Model 3 GSLT is valid at later epoch only for $\lambda_{1}=9$ and condition of thermal equilibrium satisfied for all cases of $\lambda_{1}.$
- For Sharma-Mittal entropy
  For Model 1 validity of GSLT cannot hold at later epoch as all trajectories remain in the negative phase and the thermal equilibrium condition cannot occurs for all cases, for Model 2 the stability condition of GSLT and thermal equilibrium maintained at present as well as later epoch and for Model 3 GSLT is valid only for two cases $\lambda_{1}=8,8.5$ and the thermal equilibrium condition cannot satisfied for any case.
- For Logarithmic corrected entropy
  The validity of GSLT at Hubble horizon for Model 1 confirms at later epoch and the stability condition of thermal equilibrium maintained for $\lambda_{1}=9,$ for Model 2 GSLT validity and thermal equilibrium condition confirmed at present epoch as well as later epoch and for Model 3 with the increasing value of $\lambda_{1}$ the stability condition of GSLT preserved and the thermal condition is invalid for $\lambda_{1}=8$ and the thermal equilibrium condition occured for other two cases $\lambda_{1}=8.5,9.$
- For $R\overset{´}{e}nyi$ entropy
  The stability condition of GSLT for Model 1 is satisfied for all cases at present as well as later epoch and the thermal equilibrium condition confirmed at present epoch as well as later epoch only for two cases $\lambda_{1}=8,8.5$ and the stability of thermal equilibrium condition cannot preserved with the increasing value of $\lambda_{1}.$ For Model 2 GSLT is valid for all values of $\lambda_{1}$ at present as well as later epoch and with the increasing value of $\lambda_{1}$ the thermal equilibrium condition satisfied and for Model 3 GSLT maintained the validity at later epoch for $\lambda_{1}=9$ and the stability of thermal equilibrium condition cannot satisfied for each case.

The stability of GSLT and thermal equilibrium condition for each model satisfied only for some cases at present as well as later epoch.

Physically some results are not valid as it is not possible that GSLT and thermal equilibrium condition satisfies at the same time. We shown the validity of GSLT and thermal equilibrium condition mathematically only.

## Figures and Tables

**Table 1:** First-order equations and corresponding solutions.

$\dot{\phi_{i}},i=1,2$	$\phi_{i}\left(t\right)$
$\lambda_{1}\left(1-\phi_{1}^{2}\right)$	$\tanh(\lambda_{1}t)$
$\lambda_{2}\cosh\left(\phi_{2}\right)$	$\arcsin h[\tan\left(\lambda_{2}t\right)]$

**Table 2:** Differential equations and their solutions.

$\dot{\phi_{i}},i=3,4$	$\phi_{i}^{\left(k\right)}\left(t\right),k=1,2$
	$\frac{\left(\sqrt{c_{o}^{2}-4}\right)\sinh\left(2\mu_{3}t\right)}{\left(\sqrt{c_{o}^{2}-4}\right)\cosh\left(2\mu_{3}t\right)-c_{o}},$
$-\lambda_{3}\left(1-\phi_{3}^{2}\right)+\mu_{3}\phi_{4}^{2}$	
	$\frac{\left(\sqrt{1-16c_{o}}\right)\sinh\left(4\mu_{3}t\right)}{\left(\sqrt{1-16c_{o}}\right)\cosh\left(4\mu_{3}t\right)+1},$
	where $c_{o}<-2$ and $\lambda_{3}=\mu_{3}.$
	$\frac{2}{\left(\sqrt{c_{o}^{2}-4}\right)\cosh\left(2\mu_{3}t\right)-c_{o}},$
$2\mu_{3}\phi_{3}\phi_{4}$	
	$-\frac{2}{\left(\sqrt{1-16c_{o}}\right)\cosh\left(4\mu_{3}t\right)+1},$
	where $c_{o}<\frac{1}{16}$ and $\lambda_{3}=4\mu_{3}.$

**Table 3:** Differential equations and solutions.

$\dot{\phi_{i}},i=3,4$	$\phi_{i}\left(t\right)$
	$4(\left(c_{o}^{2}\left(\lambda_{3}^{2}+4\right)-\lambda_{3}^{2}-5\right)\sinh\left(2t\right)$
$-\lambda_{3}\phi_{3}^{2}-2\phi_{3}\phi_{4}$	+$\left(c_{o}^{2}\left(\lambda_{3}^{2}+4\right)-\lambda_{3}^{2}-3\right)\cosh\left(2t\right))$
	+ $2c_{o}\sqrt{\lambda_{3}^{2}+4}{)}^{-1}.$
	$(\left(c_{o}^{2}\left(\lambda_{3}^{2}+4\right)-\lambda_{3}^{2}-3\right)\sinh\left(2t\right)$
	+$\left(c_{o}^{2}\left(\lambda_{3}^{2}+4\right)-\lambda_{3}^{2}-5\right)\cosh\left(2t\right)-2\lambda_{3})\times$
$-\phi_{4}^{2}-\phi_{3}^{2}+1$	$(c_{o}^{2}\left(\lambda_{3}^{2}+4\right)-\lambda_{3}^{2}-5)\sinh\left(2t\right)$
	+$\left(c_{o}^{2}\left(\lambda_{3}^{2}+4\right)-\lambda_{3}^{2}-3\right)\cosh\left(2t\right))+2c_{o}\sqrt{\lambda_{3}^{2}+4}{)}^{-1}.$
